# Drought Stress Responses in Soybean Roots and Nodules

**DOI:** 10.3389/fpls.2016.01015

**Published:** 2016-07-12

**Authors:** Karl J. Kunert, Barend J. Vorster, Berhanu A. Fenta, Tsholofelo Kibido, Giuseppe Dionisio, Christine H. Foyer

**Affiliations:** ^1^Department Plant Production and Soil Science, Forestry and Agricultural Biotechnology Institute, University of PretoriaPretoria, South Africa; ^2^Melkassa Agricultural Research Centre, Ethiopian Institute of Agricultural ResearchAdama, Ethiopia; ^3^Faculty of Science and Technology, Research Centre Flakkebjerg, Department of Molecular Biology and Genetics, Aarhus UniversityAarhus, Denmark; ^4^Centre for Plant Sciences, School of Biology, Faculty of Biological Sciences, University of LeedsLeeds, UK

**Keywords:** *Glycine max*, root architecture, nodule traits, soybean omics, water stress

## Abstract

Drought is considered to be a major threat to soybean production worldwide and yet our current understanding of the effects of drought on soybean productively is largely based on studies on above-ground traits. Although the roots and root nodules are important sensors of drought, the responses of these crucial organs and their drought tolerance features remain poorly characterized. The symbiotic interaction between soybean and rhizobia facilitates atmospheric nitrogen fixation, a process that provides essential nitrogen to support plant growth and development. Symbiotic nitrogen fixation is important for sustainable agriculture, as it sustains plant growth on nitrogen-poor soils and limits fertilizer use for crop nitrogen nutrition. Recent developments have been made in our understanding of the drought impact on soybean root architecture and nodule traits, as well as underpinning transcriptome, proteome and also emerging metabolome information, with a view to improve the selection of more drought-tolerant soybean cultivars and rhizobia in the future. We conclude that the direct screening of root and nodule traits in the field as well as identification of genes, proteins and also metabolites involved in such traits will be essential in order to gain a better understanding of the regulation of root architecture, bacteroid development and lifespan in relation to drought tolerance in soybean.

## Introduction

The world-wide soybean production in 2015/2016 will be 320.15 million metric tons (Global soybean production.com, 2016). Sustainability of soybean yields is, however, threatened by predicted climatic changes with persistent droughts over many parts of the world (Dai, 2013; Foyer et al., 2016). Selection of more drought-tolerant soybean cultivars is therefore required to address this imminent threat to food and protein security (Ku et al., 2013).

Recent advances in current understanding of the effects of drought on soybean growth have predominantly been based on evaluation of above-ground (shoot) traits, with flowering and seed stages particularly sensitive to drought stress. In contrast, drought effects on soybean roots, and specifically root nodules, has been less studied. Moreover, relatively little information is available concerning how drought affects the symbiotic relationship between nitrogen fixing soil rhizobia and the host plant (Ferguson et al., 2010). This unique symbiotic relationship is initiated by the plant through release of root flavonoids into the rhizosphere, recognized by compatible *Rhizobium sp.* Flavonoid signaling results in bacterial production of specific lipochito-oligosaccharides (Nod factors) secreted by rhizobia (Kondorosi et al., 2013). Nod factors are in turn recognized by specific LysM receptor-like kinases located on root epidermal cells. Nod factor binding results in genetic and metabolic signaling cascades that are mediated, at least in part, by cell specific nuclear Ca^2+^ oscillations (Charpentier and Oldroyd, 2013). The signaling cascade results in increased division of cortical cells within the root infection area with formation of composite structures derived from the two symbiotic partners (Gage, 2004). This bacterial infection thread allows rhizobia penetrating deep into the dividing cellular profile resulting in a new organ, the N-fixing ‘nodule,’ housing infected rhizobia replicating within nodule cells (Oldroyd et al., 2011; Oldroyd, 2013). Inside infected cells, rhizobia are encapsulated with a plant-derived membrane forming the facultative organelle, the symbiosome (Oldroyd, 2013). The symbiosome provides strict plant control on movement of nutrients from bacteria and regulates rhizobial activity and persistence. The symbiosis is facultative and initiated by nitrogen starvation of the host plant (Maróti and Kondorosi, 2014). Within the symbiosome, bacteria differentiate into an endosymbiotic form (bacteroids) for fixing N_2_ into ammonium. This energy-requiring process is dependent on photosynthate supplied by the shoots. Fixation is catalyzed by the bacterial enzyme nitrogenase requiring a low, but stable, oxygen environment achieved in part through activity of a nodule localized oxygen diffusion barrier. Continual oxygen flux to support bacteroid respiration is finally ensured by the nodule expressed protein leghaemoglobin.

**FIGURE 1:**
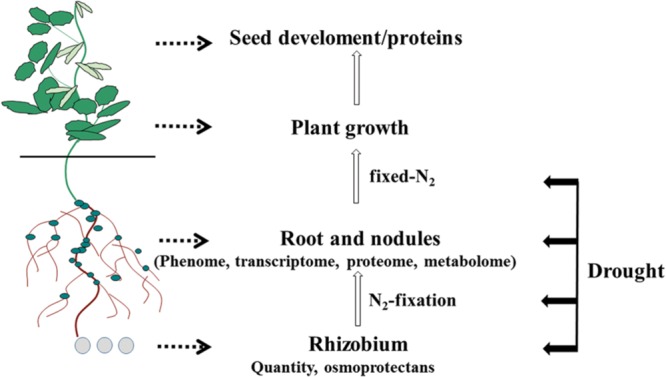
**Below-ground plant organs affected by drought that can be analyzed using omics technologies, including the rhizobia that form symbiotic relationships with soybean roots**.

**FIGURE 2:**
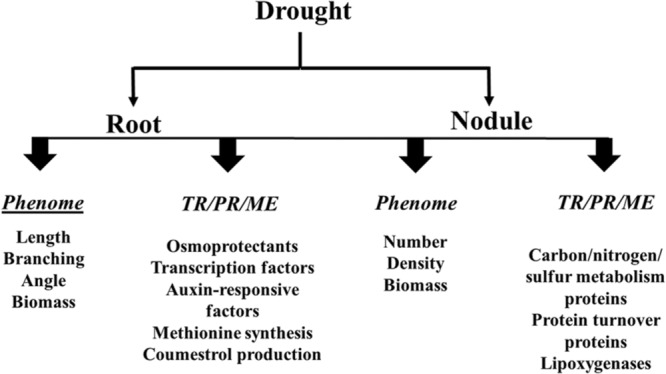
**Effects of drought on the soybean root and nodule phenome, transcriptome (TR), proteome (PR) and metabolome (ME)**.

The purpose of this mini-review is to provide an update on the recent developments that have enhanced our understanding of how drought influences soybean roots/nodules, with a particular focus on root and nodule phenome and symbiotic nitrogen fixation. Effects of drought on the soybean root/nodule transcriptome, proteome and metabolome are also outlined as illustrated in **Figures 1** and **2**.

## Drought-Induced Changes to the Root Phenome

Soybean has an allorhizic root system consisting of a primary root (tap root) and lateral (basal) roots (Ao et al., 2010; Fenta et al., 2014). Decreased root lengths and dry biomass accumulation have been reported in many soybean accessions under drought conditions (Thu et al., 2014). Drought not only changes root architecture (root depth, root branching density, and root angle) but also partitioning of root to shoot biomass with an increase in root mass (Franco et al., 2011; Fenta et al., 2014). Several studies have provided strong evidence that root types either penetrating deep into the soil and attaining greater “root mass at depth” (Lopes et al., 2011; Ali et al., 2016) or roots with large xylem diameters and/or larger lateral root systems with more root hairs are advantageous under drought conditions (Tanaka et al., 2014; Vadez, 2014). Such roots tend to have a greater total surface area, which facilitate maximal moisture and nutrient extraction to maintain photosynthesis (Blum, 2011; Lopes et al., 2011; Comas et al., 2013). The soybean cultivar Jackson is an excellent example possessing this type of root system with long roots growing deep into the soil allowing better water uptake than other more drought-sensitive cultivars (Serraj et al., 1997; Fenta et al., 2014). However, identification of soybean cultivars with improved root architecture characteristics still remains challenging. Classic root phenotyping approaches including analysis of soil cores and applying standard excavation techniques to determine root traits are still the methods of choice (Fenta et al., 2014). Future more accurate non-destructive methods under development are transparent tubes (mini-rhizotrons), to measure with a camera various root characteristics around the outside walls of the tubes, or *in situ* tomographic measurements of the root system with X-rays (Mooney et al., 2012; Eberbach et al., 2013).

## Changes in the Root Transcriptome and Proteome

Transcriptome analysis and Next-Generation Sequencing (NGS) are current strategies to particularly study plant responses to abiotic stress (Fan et al., 2013). Identification of genes underpinning root traits and related drought responses have recently received intensive interest (Manavalan et al., 2009; Libault et al., 2010; Comas et al., 2013; Thao et al., 2013; Satbhai et al., 2015). Among 3,000 genes strongly up-regulated in roots by drought were several transcription factors, receptor-like kinases, calcium signaling components as well as jasmonate and abscisic acid biosynthetic genes (Tripathi et al., 2016). Transcriptome responses to drought are also highly dependent on stress intensity and duration as well as species and organs investigated. In the case of soybean roots, 145 root genes were for example differentially expressed due to drought. Identified gene functions demonstrated a complex drought response with genes involved in different multiple biochemical pathways related to drought adaptation (Stolf-Moreira et al., 2011). Applying the deep SuperSAGE method, increased expression of 1,127 unitags in a stress-tolerant soybean accession were associated with responses to hormone stimuli, water stress, as well as oxidative stresses (Neto et al., 2013). Other transcriptome studies were carried out with soybean cultivars W82 and DT2008. The genome of W82, often used as a model cultivar, was sequenced several years ago (Schmutz et al., 2010). DT2008, an economically important soybean cultivar and widely grown in Vietnam (Vinh et al., 2010; Sulieman et al., 2015), has high drought tolerance (Ha et al., 2013; Sulieman et al., 2015) and better nodule development under drought when compared to W82 (Sulieman et al., 2015). By comparing the root transcriptomes of DT2008 and W82, seedlings under normal and dehydration conditions (2 and 10 h treatment), 38172 soybean genes, which changed in expression, could be annotated with high confidence (Ha et al., 2015). Data suggested that higher drought tolerability of DT2008 roots, when compared to W82, might be attributed to a higher number of root genes induced by early dehydration than by prolonged dehydration. The higher drought tolerability of DT2008 vs. W82 might be further attributed to differential expression of genes associated in osmo-protectant biosynthesis, detoxification, cell wall-related proteins, kinases, transcription factors as well as phosphatase 2C proteins (Ha et al., 2015). In particular, the levels of transcripts encoding the auxin responsive factors (ARFs) *GmARF33* and *GmARF50* were greatly increased in shoots and roots. For example, *GmARF50* transcripts were rapidly increased by 15- and 30-fold after 2 and 10 h of dehydration, respectively (Ha et al., 2013). Further, subjecting Williams 82 to increasing drought conditions caused the total differential expression of 6609 transcripts including many genes involved in hormone (auxin/ethylene), carbohydrate, cell wall-related secondary metabolism as well as transcription factors controlling root growth (Song et al., 2016). However, a more in-depth functional characterization is still required to determine how these transcripts will lead to better drought tolerance.

Several proteomics study have also been carried out to unravel the abiotic stress response mechanism in soybean (Hossain et al., 2013) and root proteins, changed in abundance due to drought, were involved in osmotic-stress responses (Toorchi et al., 2009). These proteomics studies also highlighted again the key role of root genes involved in osmo-protection and encoding kinases and transcription factors in the drought response. Interestingly, decreased amounts of methionine synthase were also found as a response to drought (Mohammadi et al., 2012; Oh and Komatsu, 2015). This enzyme catalyzes the conversion of cysteine into methionine in sulfur metabolism. This protein, of central importance in sulfur metabolism, might therefore be a drought responsive protein underpinning possible epigenetic controls that are triggered in drought response. Lower methionine synthase activity under drought might further negatively affect soybean growth due to less available methionine for protein biosynthesis. Furthermore, a great number of root metabolites, such as coumestrol, also change during drought (Tripathi et al., 2016). Coumestrol possibly stimulates mycorrhizal colonization and there is emerging evidence that mycorrhizal plants have improved drought tolerance (Armada et al., 2016).

## Exploring the Nodule Phenome

Soybean has determinate nodules formed by the symbiotic interaction of a soybean plant with *Bradyrhizobium* (Herridge et al., 2008). Despite symbiotic N_2_ fixation is adequate to meet the nitrogen needs of the soybean crop, high-yielding soybeans benefit from supplemental N applications, since N_2_ fixation capacities are not always sufficient to produce high yields. However, nodule numbers are only decreased when soybean plants are subjected to severe drought conditions (Fernandez-Luquen et al., 2008; Márquez-García et al., 2015). Nodule drought tolerance has been linked to the ability to sustain a supply of photosynthate to the nodules during drought and to greater nodule biomass (King and Purcell, 2001). The relationships between the frequency and intensity of nodulation and root growth and architecture are, however, still poorly understood, particularly the factors that control nodule density per unit root length in the absence and presence of stress. Furthermore, although nitrate is required for root development, it has a negative impact on nodulation (Ferguson et al., 2010). Therefore, improving root and nodule development under drought requires in the future a better understanding of the consequences of the signaling of nitrate and related nutrients, such as phosphate, on root development together with the impact of drought-induced changes on nutrient availability on symbiotic nitrogen fixation.

Exposure to severe drought also impairs nitrogenase activity. This may be caused by several factors including impairment of the supply of photosynthate to the nodules to drive symbiotic nitrogen fixation and breakdown of the oxygen diffusion barrier or loss of leghemoglobin (King and Purcell, 2006; Arrese-Igor et al., 2011). In exchange for photosynthate, soybean nodules deliver reduced nitrogen in form of ureides (allantoin and allantonic acid), mediated by UPS1 transporter proteins (Collier and Tegeder, 2012), to the plant, providing the nitrogen that is required for biomass production and finally seed protein production. However, the molecular mechanisms that support ureide export to the plant via the xylem have so far not been fully characterized.

## Exploring the Nodule Transcriptome and Proteome

Studies on nodule transcriptome profiles have largely focused on the early stages of nodule development. The release of the complete soybean genome (Schmutz et al., 2010) and the RNAseq atlas of genes expressed in fourteen different soybean tissues, including nodules, (Severin et al., 2010) provide currently a useful genetic resource to also study single nodule genes, or gene networks, after drought exposure with automated bioinformatics methods predicting also gene regulatory networks (Zhu et al., 2013). A recently predicted soybean nodulation-related regulatory gene network, consisting of 10 regulatory modules, might be also applicable to investigate drought effects on nodule gene expression. Transcriptome studies have been generally limited by poor genome annotation, but the situation is gradually improving with the growing annotated soybean genome database (Severin et al., 2010). The previous application of Suppression Subtractive Hybridisation (SSH) technology on soybean nodules, in the absence and presence of drought, largely identified sequences with unknown functions. Only relatively few drought-responsive transcripts had known functions applying this technology including ferritins and metallothionins involved in metal detoxification, particularly in response to oxidative stress (Clement et al., 2008). We recently also explored the nodule cysteine protease transcriptome during developmental nodule senescence. Several papain-like and legumain-like cysteine proteases, also called vacuolar processing enzymes (VPEs), were identified to be strongly expressed during nodule senescence (Van Wyk et al., 2014). In nodules, papain-like cysteine proteases have known functions in the regulation of bacterial symbiosis and nitrogen fixation, they target for example leghemoglobin (Van de Velde et al., 2006; Li et al., 2008). We have recently also found that inhibition of papain-like cysteine protease activity can improve soybean tolerance to drought and favors increased nodulation (Quain et al., 2014, 2015). VPEs are involved in developmental senescence and activation of pre-proteases. With their caspase-like activity, they further play an important role in programmed cell death (PCD) (Hara-Nishimura et al., 2005; Roberts et al., 2012). Other such identified cysteine proteases with caspase-1 like activity include the 20S proteasome beta subunit 1 (PBA1; casapase-3 like activity), DEVDase (Hatsugai et al., 2009; Gu et al., 2010; Han et al., 2012), YVADase (Hara-Nishimura et al., 2005), VKMDase (Bonneau et al., 2008), VEIDase, and TATDase (Chichkova et al., 2010). Cathepsin B, also with caspase-3 activity and responsible for PCD, is normally bound to an endogenous cysteine protease inhibitor but is released upon perception of PCD triggers (Ge et al., 2016). An interesting aspect would be therefore to investigate in the future if exposure to drought may compromise such protease-inhibitor interactions and hence lead to PCD.

Proteome analyses on legume nodules have not only been carried out to better understand the soybean symbiosome (Clarke et al., 2015), but also to find drought-induced proteome changes. The nodule proteomes of *Medicago truncatula* and *Glycine max* were recently compared under drought and drought caused the down-regulation of the entire nodule proteome. Particular proteins down-regulated were lipoxygenases and proteins involved in carbon, nitrogen and sulfur metabolism, similar to the root proteome, and proteins involved in protein turnover (Gil-Quintana et al., 2015). The study also highlighted a high degree of similarity between both legume proteomes. Research carried out on *M. truncatula* might be, therefore, also directly applicable to other economically important legume crops, such as soybean. Applicable findings include that drought induces a major change in the metabolic profile of *M. truncatula* nodules with accumulation of amino acids (Pro, His, and Trp) and carbohydrates (sucrose, galactinol, raffinose, and trehalose) associated with a decline of bacteroid proteins involved in C-metabolism (Larrainzar et al., 2009). Further applicable findings are that in *M. truncatula* nodules methionine biosynthesis is particularly affected by drought and that, despite sufficient S-availability, the nitrogen fixation rate in response to drought declines. Such decline is associated with a down-regulation of proteins involved in biosynthesis of methionine and *S*-adenosyl-L-methionine (SAM), a precursor in ethylene biosynthesis, as well as ethylene biosynthesis (Larrainzar et al., 2014). These results provide strong evidence for a central importance of sulfur metabolism in the drought response. Also, the recent finding of significant delay in drought-induced leaf senescence in nodulated *M. truncatula* plants with nodulated plants recovering more effectively from drought, relative to non-nodulated plants, might also be applicable to soybean (Staudinger et al., 2016).

## Focus Areas for Intensive Exploration

Technology development is key to future progress. In particular, a major focus must be more accurate, non-invasive monitoring of root architecture and nodulation in the field. Extraction of the entire root system from field-grown plants (“shovelomics”) to determine drought-induced changes in root architecture is often laborious and requires destructive root excavation (Fenta et al., 2014). Scientists are often reluctant to work in the field with such system. High throughput root and nodule phenotyping under field conditions by direct screening of root and nodule systems in the soil, without the need for excavation, is therefore very likely crucial for any future soybean improvement.

An exciting future task will also be the development of root and nodule transcriptome, proteome as well as metabolome maps in relation to drought (Nguyen, 2016). However, this should also include more in-depth functional characterization of transcripts/proteins/metabolites and how they lead to better drought tolerance. Transcriptomic and proteomics studies already indicate that up-regulation of genes involved in osmo-protection and coding for kinases and transcription factors are playing a key role in the drought response in addition to down-regulation of genes coding for proteins involved in nitrogen and sulfur metabolism. Deeper understanding of drought-induced changes in gene/protein/metabolite expression patterns will provide information on gene/protein/metabolite networks underpinning phenotypic traits relevant to stress tolerance and also how they ultimately link to phenome changes allowing new insights into changes required for drought recovery.

Improving the soybean-rhizobia symbiosis might also contribute to better drought tolerance. More robust rhizobia with better osmo-tolerance of rhizobia to persist for longer in droughted soils might thereby be a contributor (Mhadhbi et al., 2013). Recent research has also provided evidence that plant growth-promoting rhizobacterium (PGPR) improve plant adaptation to drought by stimulating lateral root formation and increasing shoot growth (Rolli et al., 2015) with stimulation partly caused by bacterium-produced volatile organic compounds (Wintermans et al., 2016). Also, salicylic acid to assemble a better root microbiome might play a role, since salicylic acid can modulate colonization of the root by specific bacterial families (Lebeis et al., 2015). Pyrrolizidine alkaloids (PAs), involved in plant cell re-programming for micro-symbiont entry, might be further a contributor and a target for investigation. A plant-homo-spermidine synthase (HSS), the first pathway-specific enzyme of PA biosynthesis, is exclusively localized in nodules (Irmer et al., 2015) suggesting that the plant is the main PA producer. Investigation how drought affects expression of soybean nodule HSS (Glyma.06g126700) might be therefore interesting.

Drought might finally also affect expression of nodule specific cysteine-rich antimicrobial peptides (NCR AMPs) essential for bacteroid development and found in legumes with indeterminate nodules (Mergaert et al., 2003; Horváth et al., 2015). In *M. truncatula* nodules, the bacteria undergo an irreversible differentiation process producing elongated polyploid bacteroids that cannot resume cell division. This differentiation process is controlled by nodule specific NCRs (Van de Velde et al., 2010; Haag et al., 2011, 2012; Frendo et al., 2013; Horváth et al., 2015). Although 138 NCRs were recently detected in *M. truncatula* bacteroids (Durgo et al., 2015) such NCRs, or peptides with similar antimicrobial functions, have so far not been found in soybean. Search for similar peptides in soybean and characterizing them under drought might be therefore an interesting future task.

## Author Contributions

KK has overall organized the paper and has written the draft paper. BV contributed with knowledge about proteolytic events in nodules and transcriptome analysis. BF contributed with his knowledge about root architecture, nodule characterization and recent developments in root and nodule screening. TK contributed with her knowledge about rhizobia screening for drought tolerance. GD contributed with his knowledge about legumains. CF contributed with her knowledge about nodule biology and and was involved in final writing of the paper.

## Conflict of Interest Statement

The authors declare that the research was conducted in the absence of any commercial or financial relationships that could be construed as a potential conflict of interest.
